# Benefits of Explorative Saccade Training in Patients with Advanced Glaucomatous Visual Field Defects—A Randomized, Placebo-Controlled Study

**DOI:** 10.3390/jcm14092876

**Published:** 2025-04-22

**Authors:** Nawfel Ferrand, Susanne Trauzettel-Klosinski, Gunnar Blumenstock, Bogomil Voykov, Stephan Kuester-Gruber

**Affiliations:** 1 Centre for Ophthalmology, University of Tuebingen, 72076 Tuebingen, Germany; bogomil.voykov@med.uni-tuebingen.de; 2 Vision Rehabilitation Research Unit, Centre for Ophthalmology, University of Tuebingen, 72076 Tuebingen, Germany; susanne.trauzettel-klosinski@uni-tuebingen.de (S.T.-K.); stephan.kuester@uni-tuebingen.de (S.K.-G.); 3 Department of Clinical Epidemiology and Applied Biometry, University of Tuebingen, 72076 Tuebingen, Germany; gunnar.blumenstock@med.uni-tuebingen.de

**Keywords:** glaucoma, scotoma, eye movements, explorative saccade training, quality of life, reading training

## Abstract

**Purpose**: Patients with advanced glaucoma have visual field defects that impair mobility and quality of life (QoL). We aim to determine the effects of exploratory saccade training (EST) in such patients with bilateral overlapping scotomas that affect at least one visual field quadrant. **Patients and Methods**: This study was approved by the Ethics Committee of the Medical Faculty of the University of Tuebingen, Germany, and was registered in the German Clinical Trials Register (DRKS DRKS00031082, date of approval: 2 February 2023). We randomly assigned 27 patients to two groups, one of which trained with a computer-based EST (group 1). A control group (group 2) first used reading training (rapid serial visual presentation, RSVP, a single-word presentation to minimize eye movements) as placebo training (PRT) in regard to EST, which trains eye movements and, in a later phase, also used EST. Each training method required 6 weeks of home training. Main outcome variables were reaction time (RT) during the EST training sessions, RT during a natural search task (table test), reading speed (RS) during training on the screen, and during reading printed paragraphs aloud. QoL was assessed by a questionnaire. **Results**: Reaction times during EST and the table test improved significantly, which indicated transfer of the training effect to daily life. RS and QoL were reduced at baseline. Reading training improved RS significantly and reached normal median values. QoL improved significantly in the sub-categories regarding mobility problems in group 1. Patients with inferior field defects were more impaired and improved more than those without inferior field defects. **Conclusions**: As a supplement to the necessary treatment for glaucoma, EST is an effective home training method for rehabilitation by improving reaction time in daily living tasks for patients with advanced glaucoma. Reading training improved RS while reading from a screen as well as reading printed text.

## 1. Introduction

Glaucoma is the most common cause of irreversible blindness and the second most common irreversible cause of severe and moderate vision impairment worldwide [1]. Patients with glaucoma are often not aware of their visual impairment, which is perceived only in the late stages of the disease, due to its slow and painless loss of peripheral visual field. In addition, this is supported by the plasticity of the visual cortex, which masks scotomata [2]. This is why an estimated 90% of glaucomas are diagnosed too late in low- or middle-income countries, the figure being 50% in high-income countries [3].

However, early detection and effective therapy are essential in glaucoma to preserve patients’ quality of life, since visual loss in glaucoma is irreversible.

Currently, intraocular pressure (IOP) is the only modifiable risk factor for glaucoma progression [4]. However, IOP-lowering treatments can only slow down or stop the progression of the disease, but they cannot restore the visual field. Consequently, there is an unmet need to develop strategies to improve quality of life (QoL) of patients with advanced visual field defects. Furthermore, existing therapies have numerous side effects, which also impact compliance with the treatment.

As with many other diseases that restrict the visual field, vision rehabilitation approaches have shown substantial benefits. We showed in patients with hemianopia, in adults and in children, as well as in patients with retinitis pigmentosa, significant improvement of visual search on a screen, in daily living tasks and QoL [5,6]. The goal of these methods is not to reduce the size of the scotoma, but to support compensatory mechanisms by scanning eye movements that can improve visual perception by utilizing the complete field of gaze [7] and thus QoL.

In this study, we aimed to determine the effects of exploratory saccade training (EST) in patients with glaucoma with bilaterally overlapping absolute or nearly absolute scotomas. We particularly focused on the reaction times (RT) during the EST training sessions and during a natural search task, as well as on reading performance and QoL based on a self-assessment questionnaire.

Reading training was applied as a placebo training in regard to the EST—using single words on a screen (rapid serial visual presentation, RSVP) in order to minimize eye movements—in contrast to the eye movement training in EST. In addition, we were interested in testing whether reading training might improve reading performance in patients with glaucoma. A number of studies have demonstrated impaired reading in patients with glaucoma [8,9,10,11]. Therefore, we also investigated reading ability and the potential for its improvement by reading training in our present study. In previous studies in patients with macular scotoma, we and other authors found significant improvement of reading speed (RS) caused by RSVP reading training [12,13,14].

To the best of our knowledge, explorative saccade training and reading training have not been applied in patients with glaucoma before.

We hypothesize the following:

(1) EST improves RT on a screen;
(2) EST improves RT in a natural search task;
(3) PRT does not influence RT in search tasks;
(4) PRT does not influence RS.

## 2. Patients and Methods

### 2.1. Patients

This study was conducted according to the guidelines of the Declaration of Helsinki [15] and approved by the Ethics Committee of the Medical Faculty of the University of Tuebingen, Germany (protocol code 804/2022B02 and date of approval 31 January 2023), and was registered in the German Clinical Trials Register (DRKS DRKS00031082, date of approval: 2 February 2023). We reviewed the charts of all patients who presented in the glaucoma clinic at the University Eye Hospital Tübingen between October 2022 and March 2023 and had had a visual field test within the last 6 months. Based on these findings, we defined the following inclusion and exclusion criteria:

Inclusion criteria were the following: A diagnosis of glaucoma (clinically and confirmed by the use of topical agents for glaucoma therapy or surgery for glaucoma in the past), age over 18 years, minimal visual acuity of 0.4 (20/50, 0.4 logMAR) in the better eye, perimetry during the last 6 months, and the bilaterally overlapping absolute scotomatous area had to exceed the size of a quadrant in the central 30 degrees of the visual field. Patients were considered as functionally monocular if perimetry was not possible because their vision was too impaired, or if visual acuity in one eye was worse than 0.05 (20/400, 1.3 logMAR). In these cases, only the perimetry of the better eye was considered for inclusion.

Exclusion criteria were the following: Bilaterally overlapping scotomata smaller than a quadrant, comorbidities causing additional visual field defects, planned or emergency eye surgery in the last 6 weeks, diabetic retinopathy, advanced neovascular age-related macular degeneration (AMD) or uveitis because of the possible dependence of visual acuity on the primary disease. Initial stages of cataract or mild dry AMD were not considered exclusion criteria.

In addition, we also excluded patients without any computer experience or with cognitive and/or motor impairment, which could prohibit the usability of the computer-based eye movement training.

In total, 1178 patients of our glaucoma consultation were screened. Only 83 patients matched the inclusion criteria and were eligible. A further 56 patients were excluded because of the following reasons: planned glaucoma operation in the near future, no consent to participate, distance of residence to hospital too far, lacking computer knowledge or motivation.

**Figure 1:**
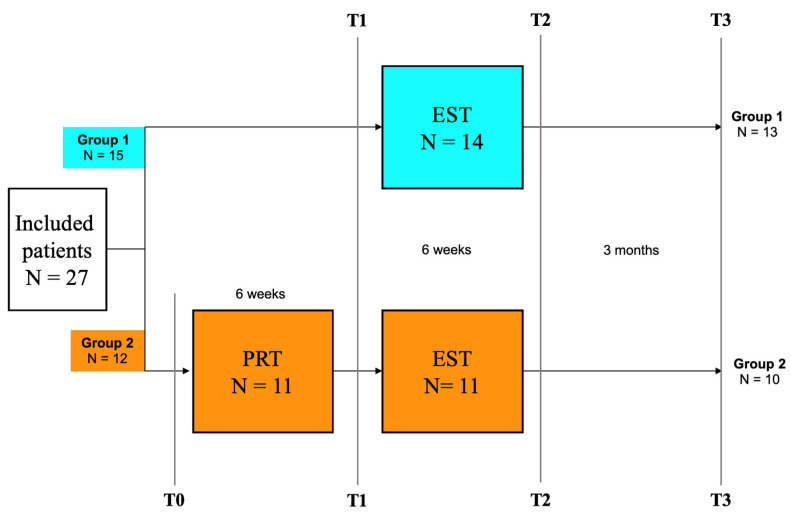
Simplified flow chart of the study design: group 1 (only EST) and group 2 (first PRT, then EST). T0: (only group 2) start of PRT, T1: (both groups) start of EST, T2: after EST, T3: follow-up after 3 months. PRT: placebo reading training, EST: explorative saccade training.

Ultimately, 27 participants were recruited and randomized (see Figure 1). During the study, one patient of each group dropped out between T1 and T2: in group 1, one patient complained about pain in the wrist during the training and had to be treated because of carpal tunnel syndrome and could not complete the training. In group 2, one patient changed his mind and withdrew from the study because of lack of time. At T3, one patient of each group did not respond to the questionnaire.

Altogether, the patients’ collected clinical data includes age, sex, past history of glaucoma, types of glaucoma, IOP at the beginning of the study, IOP-lowering medication, prior surgical procedures and best corrected visual acuity (BCVA). In addition, data on the native language, the education status and the reading habits of all patients were collected.

### 2.2. Perimetry

Visual field testing was performed using the Octopus 900 perimeter with the 30-2 dynamic standard automated strategy white/white, III (Haag-Streit AG, Koeniz, Switzerland). In two patients with low vision, 90° semi-automated kinetic perimetry was performed. All visual field tests were analysed independently by two experienced glaucoma specialists and the decision for inclusion in the study required consensus.

An “absolute” scotoma was defined as the lowest three levels of the Visual Field Index scale with a difference to norm of at least 71% [16]. The area of the binocularly overlapping absolute scotoma had to exceed the size of a quadrant and was defined as the “blind area”. The residual area was defined as “seeing”. The blind and seeing areas were categorised using the four quadrants (superior left, inferior left, superior right, inferior right) in order to allow a correlation with the results of the EST program and the table test for the individual patient. In one patient with only residual central vision, all four quadrants were categorized as blind.

### 2.3. Study Design

To assess the specific effect of the EST, all patients were randomly assigned to either of two groups (see Figure 1): Group 1 started at T1 with EST for 6 weeks and performed only one training method. Group 2 performed two training methods, one after the other: They started at T0 with a reading training program as a placebo training (PRT) in regard to EST. After using PRT for 6 weeks, these patients started training with the EST program at T1 for another 6 weeks.

In both groups, reading speed during reading printed text (RS-print) and QoL were assessed at each visit. After a follow-up time of three months, a self-assessment questionnaire was sent to the participants to assess their QoL. Furthermore, we asked if they were still using the EST. The study compared four time points: T0, T1, and T2 were visits in the Eye Hospital, while T3 was feedback gathered from the patients while at home. A flow chart of the study design is represented in Figure 1.

### 2.4. Outcome Variables

#### 2.4.1. Reaction Time (RT) During EST on a Computer Screen

The computer-based EST program aimed to improve the reaction time (RT) to find a target during visual search in the scotomatous area while using the entire 30° visual field, which is most important for navigation [17]. Patients were instructed to train 2 × 30 min, 5 days a week, during 6 weeks. A customized software prototype (Version 1.0) was developed and used exclusively for research purposes within this study. While based on the same algorithmic principles and core functionalities as VISIOcoach—a CE-marked medical device (Class I, MDR 2017/745) developed and marketed by Odilia Vision GmbH (Radolfzell am Bodensee, Germany)—the version applied in this study was adapted for investigational use in the context of glaucoma-related visual field loss. The investigational version is not CE-marked, was not used for clinical decision-making, and was applied strictly within a controlled research framework, adhering to applicable ethical standards and relevant regulations. This software program was used to generate a random array of symbols distributed with equal probabilities in the four quadrants on the computer screen. This program was based on the training program which we previously used in studies on hemianopic patients [5,18,19] and retinitis pigmentosa [5].

**Figure 2:**
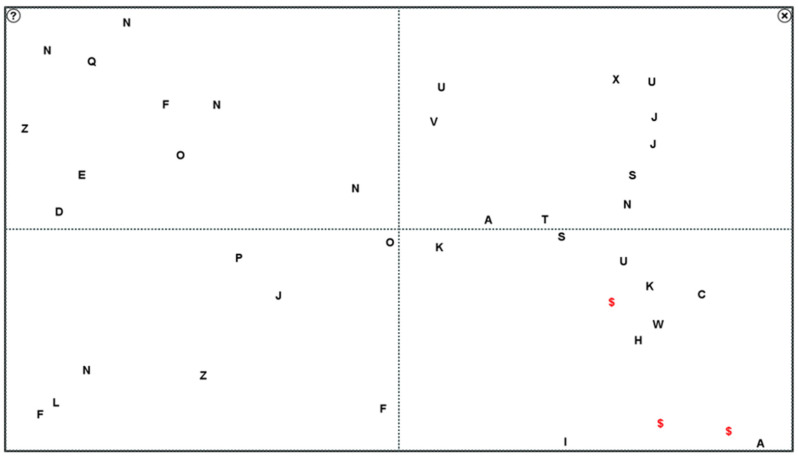
Screen shot of EST during a training session of middle level. The three “$” in the inferior right quadrant were correctly found letters by the patient. The separation into four quadrants were not shown during the training and served only for statistical analysis.

The program had three levels of difficulty: the easy level showed the digits 0–9, the middle level single capital letters A–Z, and the last level the double digits 00–99, all displayed in 12-point Arial (see Figure 2).

Patients had to search for a certain number of identical digits among others that were used as distractors, and to click on each of them with the mouse-controlled pointer. The patients were instructed to avoid head movements. In order to have fixed standardised visual field areas, the patients sat at a predefined distance to the computer screen: this distance was calculated depending on the width of the patient’s computer monitor.

The data collected during the training sessions were automatically recorded by the software on the patients’ computers. At the end of the 6 weeks of training, the saved data were gathered and analysed.

In order to avoid daily variations and possible outliers, the median of the RT of the first and last three training days at each level of difficulty was calculated. During the analyses of the RT, the screen was divided into quadrants (superior left + right, and inferior left + right, see Figure 2). A distinction between seeing and blind visual field quadrants was defined for each patient according to his or her perimetry results.

#### 2.4.2. Reaction Time (RT) in a Natural Search Task

A “table test” [6,19] was performed to measure the RT of visual search for common objects in an everyday life situation before and after the training. During this semi-quantitative table test, patients sat at a predefined distance in front of a table on which a box of 75 cm × 60 cm was placed, the contents of which were hidden by a curtain. Each box contained 30 objects of everyday life of different sizes and colours in the 30° visual field. The examiner (NF) held up a duplicate object above the middle of the closed box and observed the patient’s direction of gaze in the centre of the visual field. The patients had to close their eyes while the investigator opened the curtain. Then the patients were asked to open their eyes and to find and point at the same object on the table by only using eye movements. As soon as the matching object was pointed at, the elapsed time was measured by a stop watch, and the investigator assured that the participant closed their eyes again. The table test was conducted always by the same investigator to minimize individual manual errors. The test was performed before and after each training period (T0, T1, T2).

Corresponding to the seeing and blind quadrants that were identified by perimetry for each patient, the RT was analysed separately for the seeing and blind quadrants.

#### 2.4.3. Reading Performance

The reading training program presents single words in the centre of the screen (Rapid Serial Visual Presentation, RSVP) to minimize eye movements. Thus, the reading training served as a placebo (Placebo Reading Training, PRT) opposed to EST, where the task was to perform exploratory eye movements. The patients had to look straight to the centre of the screen. They could set the presentation of each single word on their own by pressing the key board after each word had been read (patients had to press either the space bar or right arrow key to move forward in the text, and to click the left arrow key to move backwards).

While the reading training was primarily applied as a placebo training opposed to EST, in addition, we were also interested in their reading performance itself. Since reading is one of the 15 items of the “Glaucoma Quality of Life-15” (GQL-15) questionnaire [20] and one of the most important functions that determine the QoL of low-vision patients, we measured RS with printed texts (RS-print) in **all** patients and additionally on the computer screen during the reading training (RS-screen) in the participants of group 2. We used the German version of the International Reading Speed Texts (IReST [21]) to measure RS at each visit, because it was the native language of 25 of our patients. RS was measured in words per minute (wpm), and we applied a correction factor for patients over 60 years of age, who were likely to be 9% slower [22]. In group 2, we measured RS on the computer screen during the reading training (RS-screen) and additionally while they read a printed paragraph aloud (RS-print) at T0, T1 and T2 to determine whether the use of the reading training program had caused an improvement of RS. Two patients of group 2 had to be excluded from the analysis of RS, because their RS values were extreme outliers (one patient was extremely low due to former analphabetism, the other showed values highly above normal). Furthermore, we correlated RS-screen with RS-print in order to test for potential transfer to daily life.

In group 1, who had not performed any reading training, we also measured RS-print at T1 and T2, which served as a control condition for group 2.

#### 2.4.4. Quality of Life—A Secondary Outcome Variable

A self-assessment questionnaire “Glaucoma Quality of Life-15 (GQL-15)” [20] was used in its German version [23,24] to assess a potential impact of glaucoma on daily life of our patients before and after EST. This glaucoma-specific questionnaire allows a realistic evaluation of the impairment in daily life, because eight of the fifteen questions focus on peripheral visual field defects [23] and have been used as an indicator of severity [25,26]. This questionnaire uses a scale ranging from a minimum of 15 points, indicating no subjective impairment due to glaucoma, up to a maximum of 75 points in patients with maximal visual disability and the worst QoL. Patients’ motivation to continue with the training after the end of the demanded training period due to subjective benefit of EST was assessed after a follow-up of three months.

### 2.5. Statistics

All analyses were performed using SPSS Software (IBM Corp. Released 2022. IBM SPSS Statistics for Windows, Version 29.0 Armonk, NY, USA: IBM Corp).

For descriptive statistics, we present medians and interquartile ranges (IQR). To test the normality of distributions, quantile-quantile-Plots (Q-Q plots) were created, and Shapiro–Wilk tests were performed. For most of the data, deviation from normality was confirmed (*p* < 0.05), which necessitated the use of non-parametric statistical methods. The significance level was set at α = 0.05 for all tests. We did not perform a Bonferroni correction, because it was not indicated (“closed testing procedure”) [27].

To examine within-subject differences in QoL over the four time points, we used the Friedman test, a non-parametric version of the repeated measures ANOVA. This approach was chosen for its compatibility with the repeated measures design of the study and its capacity to handle data that do not meet the normality criteria.

We used the Mann–Whitney U test to compare between-subject differences in RT of the table test and RT of the three levels of training, RS-print, and RS-screen of the placebo reading training group 2.

We used the Wilcoxon signed-rank test for assessing within-subject changes in RT of the table test and of the three levels of training pre- and post-training, RS-print, and RS-screen of the placebo reading training group 2.

The results from the analyses are reported with test statistics, *p*-values, and, where applicable, effect sizes to provide insights into the magnitude of observed effects. We calculate the effect size (Cohen’s d [28]) for the Wilcoxon signed-rank test for the table test as the Bravais–Pearson correlation coefficient: r = $\frac{Z}{\sqrt{n_{z+n_{y}}}}$. We used both Pearson’s r and Spearman’s rho correlation coefficients to test linear relationships between the variables RS-print and the RS-screen as well as RT between the table test and RT during the EST. The values of RT in the table test and during EST were not normally distributed, which necessitated the use of the Spearman rho correlation coefficient for analysis. Conversely, the Pearson correlation coefficient was used to test the linear relationship between RS-print and RS-screen at the start and end of the reading training.

The GQL-15 questionnaire offers the possible answer “I do not do this for other reasons”, which results in a point 0 (zero), which can skew the data by artificially lowering the QoL. Thus, we used imputation using the mean of the time series for this kind of missing values. Missing values in the G-QoL-15-Questionnaire were replaced by the mean, as >75% of the scale values were available [29].

To compare our patients’ QoL score at baseline with a normal sample, we compared it with the QoL score of the 95 control subjects of Tripathi et al. [30]. This procedure was also applied to compare reading speed with age-matched controls in a previous study of ours that used the same methods [22]. To perform these comparisons, we conducted an independent-samples *t*-test using the summary data of each group (means, standard deviations and number of subjects in each group). We used software by Prism 10.1.2 GraphPad [31] to perform these calculations.

## 3. Results

We use the term “significant” to mean statistically significant for better readability of the text. The values indicating the degree of statistical significance are shown only in the tables.

### 3.1. Baseline Characteristics

- **The groups**

Baseline characteristics of the patients are presented in Table 1.

Open-Angle Glaucoma (OAG) was the most frequent type of glaucoma with 22/27 (81.5%). Of these patients, 13/27 (48.1%) had already had glaucoma surgery: 7/27 (25.9%) Preserflo, 4/27 (14.8%) XEN, 2/27 (7.4%) trabeculectomy and one patient with Ahmed glaucoma valve implant

Group 1: Median visual acuity was 0.8 (IQR = 0.63–1.0). Seven patients were pseudophakic in at least one eye. The median of the mean defect values (MD) was 15.2 dB (IQR = 11.9–19.3).

Group 2: Three patients had cataract surgery in at least one eye. The median visual acuity was 20/20 (equivalent 1.0, IQR = 0.8–1.0). The median of the perimetric mean deviation (MD) of the baseline visual field in the better eye was 14.1 dB (IQR = 10.4–15.7)—see Table 1.

### 3.2. Reaction Time (RT) During Explorative Saccade Training (EST)

**Figure 3:**
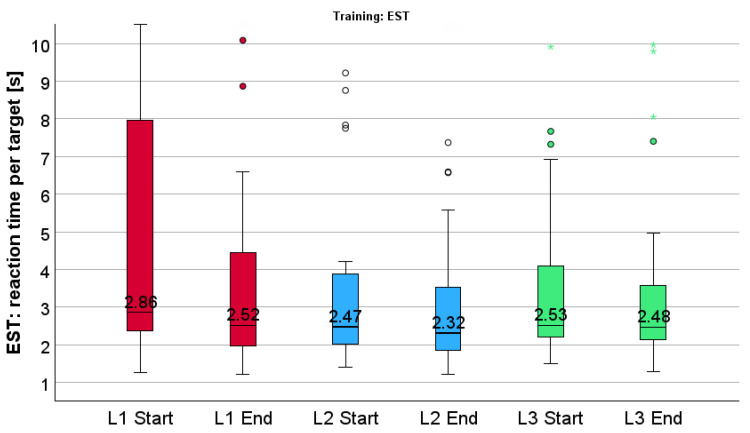
Reaction time (RT) during explorative saccadic training (EST) for all patients—level 1 (red), level 2 (blue), level 3 (green) at start and end—significant improvement on each level of difficulty. Mild outliers are depicted as circles (1.5–3.0 × IQR from the quartiles), extreme outliers as asterisks (>3.0 × IQR), following SPSS conventions.

In each group, the RT during EST improved significantly at level 1 and 2, but not at level 3, i.e., the effect remained stable. Considering all patients together, the RT improved significantly at all levels, although the improvement at level 3 cannot be considered clinically relevant, but the main point here is that the effect remains stable; see Figure 3, and for statistical values see Table 2.

For comparison between the two groups, we used the Mann–Whitney U-test at all different levels and no significant difference could be found at the six defined timepoints—see Table 2, last row.

### 3.3. Table Test

The table test was performed twice by group 1 (at T1 and T2) and three times by group 2 (at T0, T1 and T2). Between T1 and T2, both groups trained with EST. Between T0 and T1, group 2 trained with PRT, so that a significant improvement of RT from the table test was not expected.

The fact that patients fixated the centre of the table (visual field) during this test before each trial allowed further distinction between seeing and blind area. Each patient’s static visual field was classified in a seeing and a blind visual field area, and the median RT for the different quadrants was analysed separately.

**Figure 4:**
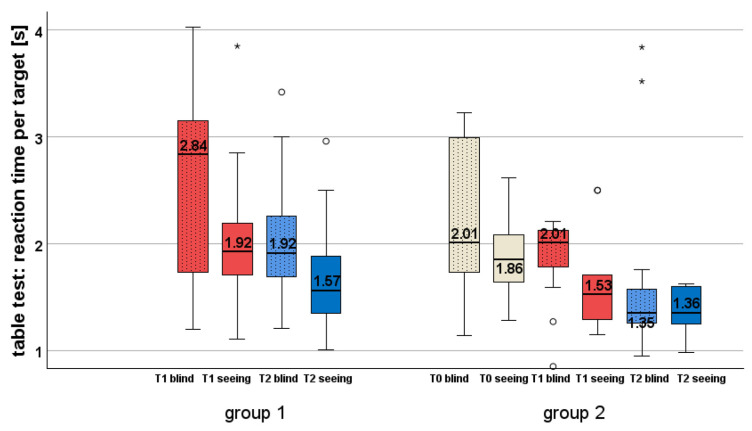
Reaction time during the table test at T0, T1 and T2 (blind/seeing). Both groups improved significantly during EST (T1–T2). Group 1 improved RT significantly in blind and seeing areas through EST, group 2 only in the blind area. During PRT, group 2 improved RT significantly only in the seeing area. Mild outliers are depicted as circles (1.5–3.0 × IQR from the quartiles), extreme outliers as asterisks (>3.0 × IQR), following SPSS conventions.

- **Within-group comparisons (see Table 3 and Figure 4)**

Group 1 significantly improved its RT per target in the blind area before (T1) and after explorative saccade training (T2) by 0.92 s (32.4%). In the seeing area, the RT improvement of 0.36 s (18.6%) per object was also significant—for detailed statistical values, see Table 3.

In group 2, RT in the blind area did not change during reading training between T0 (2.01 s) and T1 (2.01 s), but improved significantly by 0.66 s (32.8%) from the median 2.01 s (T1) to 1.35 s (T2) during EST. For the seeing area, there was a significant improvement by 0.33 s (17.7%) between T0 and T1, but not between T1 and T2—see Table 3.

**Table 3:** Reaction time of group 1 and 2 during the table test at T0, T1 and T2 (median, IQR, Wilcoxon signed-rank test and Cohen’s d).

		T0	T1	T2
group 1	blind area	-	2.84 s (1.66−3.24)	1.92 (1.67−2.27)
	Wilcoxon	-	Z = −2.105, *p* = 0.035	
	Cohen’s d	-	0.40 (medium effect)	
	seeing area	-	1.93 s (1.65−2.36)	1.57 (1.34−1.89)
	Wilcoxon	-	Z = −2.480, *p* = 0.013	
	Cohen’s d	-	0.47 (medium effect)	
group 2	blind area	2.01 s (1.73−3.23)	2.01 s (1.59−2.21)	1.35 s (1.19−1.76)
	Wilcoxon	Z = −1.689, *p* = 0.091		-
		-	Z = −2.758, *p* = 0.006	
	Cohen’s d	-		
		-	0.59 (large effect)	
	seeing area	1.86 s (1.58−2.22)	1.53 s (1.28−1.91)	1.36 s (1.23−1.61)
	Wilcoxon	Z = −2.701, *p* = 0.007		-
		-	Z = 1.376, *p* = 0.169	
	Cohen’s d	0.60 (large effect)		-
		-	-	

**Figure 5:**
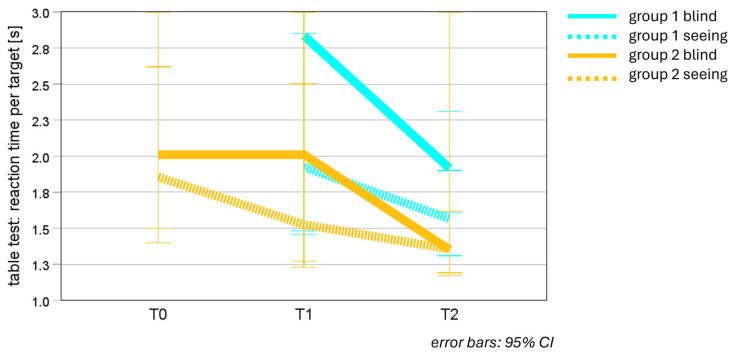
Median reaction time (RT) in table test at T0, T1 and T2—groups 1 and 2, seeing and blind areas. RT of group 1 improved significantly in the blind and seeing areas, the improvement being more pronounced in the blind visual field. RT of group 2 stayed stable after reading training as placebo (PRT) and decreased significantly in the blind area after EST. RT of group 2 improved significantly in the seeing area after PRT, but not significantly in the seeing area after EST.

- **Between-group comparisons (see Table 4 and Figure 5)**

The comparison of RT per target between the two groups at T1 and T2, i.e., before and after EST, showed no significant differences, neither in the blind, nor in the seeing area of the visual field—for statistical values, see Table 4.

**Table 4:** Between-group comparison of reaction times during table test at T1 and T2 in blind (grey) and seeing (white) areas.

Blind	Group 2 at T1	Group 2 at T2
Seeing		
**Group 1 at T1**	U = 62.5, *p* = 0.434	-
	U = 41.5, *p* = 0.096	
**Group 1 at T2**	-	U = 42.0, *p* = 0.058
		U = 50.5, *p* = 0.259

- **Correlations between RT during EST vs. RT during the table test.**

Reaction times during the EST training were highly positively correlated with RT in the table test at T1 and T2 at a significant level, which indicates a strong positive association between the two variables—for statistical values, see Table 5.

**Table 5:** Spearman’s rank-order correlation for reaction time before and after EST (level 1 start = T1 and level 3 end = T2) vs. reaction time during the table test.

Spearman’s Rank-Order Correlation		RT Table Test			
		T1		T2	
		Blind	Seeing	Blind	Seeing
**RT EST**	Level 1 start	**Rho = 0.674** *p* < 0.001, n = 24	**Rho = 0.771** *p* < 0.001, n = 23	-	-
	Level 3 end	-	-	**Rho = 0.610** *p* = 0.001, n = 25	**Rho = 0.630** *p* < 0.001, n = 24

### 3.4. Reading Speeds (RS-Print and RS-Screen)

Two different reading speeds were considered: a reading speed while reading printed paragraphs (RS-print) and a reading speed while reading on a computer screen using the rapid serial visual presentation (RSVP) method (RS-screen).

#### 3.4.1. Reading Speed During Reading Printed Paragraphs (RS-Print)

RS-print was at baseline 145.40 wpm for group 1 and 144.56 wpm for group 2 at T0. This is significantly reduced compared to the normal value of 167 wpm (SD ± 31) in elderly subjects [22]. Altpeter et al. measured a mean RS-print of 167 ± 31 wpm in 30 normally-sighted elderly persons (mean age: 64.5 (SD ± 7.2; age range 51–81 years)) under the same conditions as in the present study.

Group 1 at T1 (*n* = 13) had a median RS of 145.40 ± 52.04 wpm, which is significantly reduced compared to normal values: t (41) = 2.3512, *p* = 0.024. It was still significantly reduced at T2: t (41) = 2.3710, *p* = 0.023. However, it reached normal values at T3.

Group 2 at T0 (*n* = 10) had a median RS of 144.56 ± 93.95 wpm and at T1 (*n* = 10) of 161.68 ± 103.34 wpm: this was significantly reduced at T0 compared to normal values: (t (38) = 2.6534, *p* = 0.012, and still at T1: t (38) = 2.2445, *p* = 0.031, but no longer at T2: t (38) = 1.8665, *p* = 0.070.

**Within-group comparisons**—for detailed statistical values, see Table 6.

Patients of group 1 had 145.40 wpm at T1 and 148.11 wpm at T2 after EST. There was no significant change of RS-print between these two timepoints.

**Figure 6:**
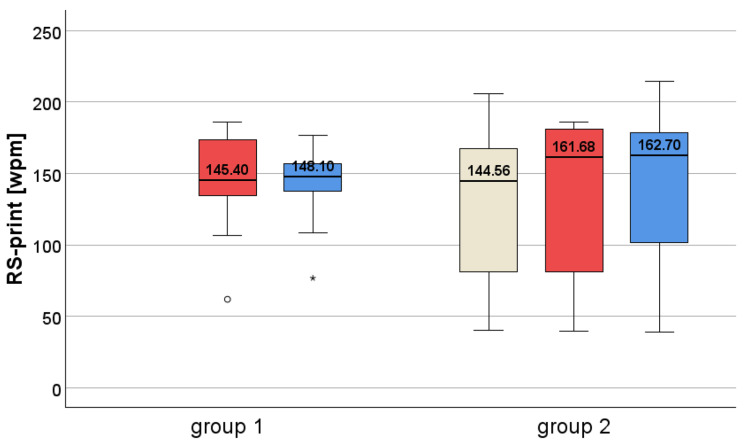
Reading speed (RS-print) while reading printed paragraphs—group 1 at T1 and T2, group 2 at T0, T1 and T2. RS-print improved in group 2 during PRT, but not during EST in either group.

Patients of group 2 had 144.56 wpm at T0 (before the PRT), then 161.68 wpm at T1, and 162.70 wpm at T2, which latter values are considered normal values of RS in elderly subjects [22]. The improvement by 17.12 wpm is considered as clinically relevant and reached significance only at T2, but not at T1. For detailed statistical values, see Table 6. Based on clinical experience and our previous studies (e.g., [13,32], we consider an improvement of RS by ≥10 wpm clinically relevant (Figure 6).

#### 3.4.2. Reading Speed from a Computer Screen (RS-Screen) During PRT

**Figure 7:**
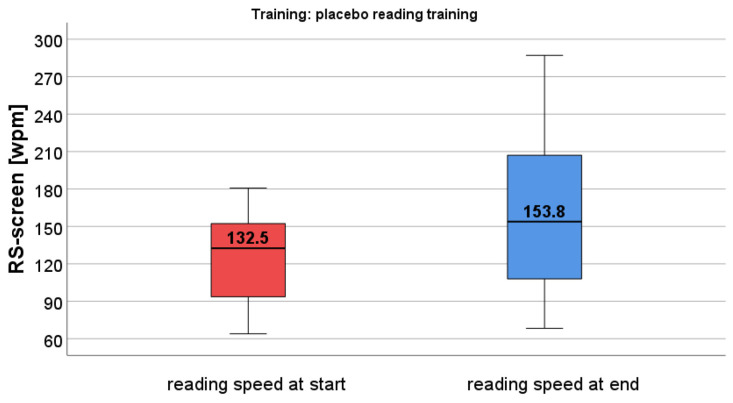
Reading speed (RS-screen) during reading training from the screen as a placebo (PRT) at the start and end of training: Group 2 improved their reading speed significantly. Correlations between RS-print vs. RS-screen during reading training at the start and end. RS-print showed a high positive correlation with RS-screen: at T0 before PRT rho = 0.757, *p* = 0.007 and at T1 (after T1) rho = 0.775, *p* = 0.05. This shows that reading training on a screen can be transferred to everyday life.

In addition to the measure of RS-print in all patients, the PRT in group 2 between T0 and T1 allowed measuring of RS-screen. RS-screen was 132.5 wpm (93.6–156.7) at the start of PRT and 153.8 wpm (96.0–212.0) at the end of PRT. Between the start and end of training, PRT also improved RS-screen significantly from 132.5 wpm to 153.8 wpm (Z = 2.312, *p* = 0.021) (Figure 7). 

### 3.5. Quality of Life

**Figure 8:**
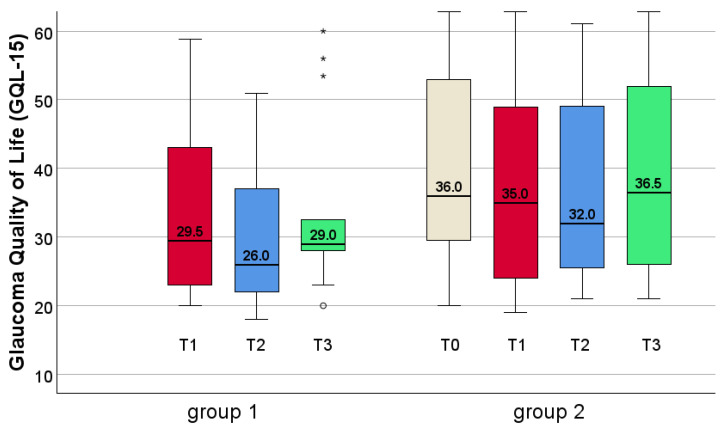
Quality of life (QoL) at T1 (beginning of EST in both groups), T2 and T3. There was no statistically significant change of QoL between the different timepoints, nor a significant difference between the groups. T0 was only assessed in group 2. Mild outliers are depicted as circles (1.5–3.0 × IQR from the quartiles), extreme outliers as asterisks (>3.0 × IQR), following SPSS conventions.

Considering the patients of group 1, the QoL was 29.5 before the eye movement training, 26.0 at T2, and finally 29.0 (without significance) after 3 months (see Figure 8). Patients of group 2 had a QoL of 36.0 at T0. After 6 weeks of placebo reading training, the QoL was 35.0 at T1, then 32.0 at T2, and finally 36.5 after follow-up 3 months later, but without significance (see Figure 8).

Considering all patients together, the QoL was 31.0 at T1 (before EST), at T2 it was 27.0, and it reached 30.0 at follow-up 3 months later. At T0 (group 2 only) the QoL was 36.0. There were no significant differences, neither by within-group comparisons (T1-T3, Friedman test, χ^2^(2) = 3.489, *p* = 0.175), nor by between-group comparisons.

There was no significant change of QoL between the three timepoints in group 1 (Friedman test, χ^2^(2) = 2.000, *p* = 0.368) nor in group 2 between the four timepoints (Friedman test, χ^2^(3) = 4.781, *p* = 0.189).

Comparing group 1 and 2 did not show significant differences between the groups at T1 (Mann–Whitney U-Test, U = 89.50, *p* = 0.501), nor at T2 (U = 98.50, *p* = 0.244), nor at T3 (U = 77.00, *p* = 0.483).

The QoL scores for the patients in the present study differed significantly from normal values [33]. Considering all patients at T1, the difference was t(118) = 8.4645, *p* < 0.001. Group 1 at T1 also showed significant different values (t(107) = 6.9504, *p* < 0.001). This was also the case for group 2 at T0 (t(104) = 9.2167, *p* < 0.001) and at T1 (t(104) = 7.6259, *p* < 0.001).

- **Patients categorized based on whether the inferior visual field was affected.**

**Figure 9:**
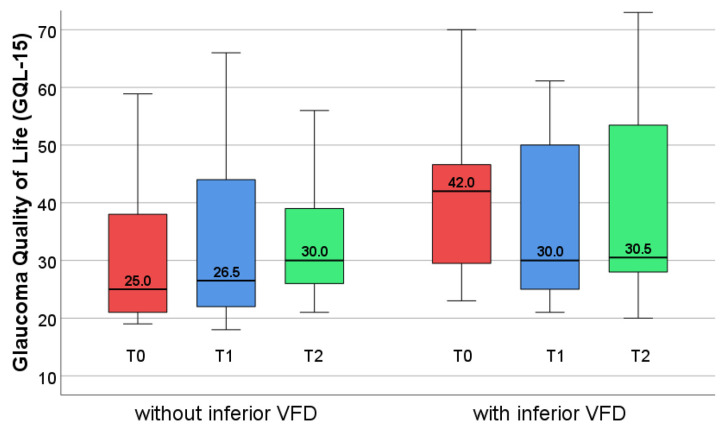
Quality of life of patients with and without inferior visual field defects. In patients without inferior defects, the score did not change between the time points. Patients with inferior field defects are much more impaired compared to those without, but this difference was not significant. They show a stronger improvement between T1 and T2—not reaching significance but reaching approximately the values of the patients without inferior field defects.

We especially focused on patients with bilaterally overlapping defects in the inferior visual field because of its importance in everyday life: three of the 11 patients in group 1 (27.3%) and eight of the 14 patients in group 2 (57.1%) were involved. The QoL of patients with an inferior blind area improved, after EST, by a median value of 12 points. Even though this change was not significant, it shows that the patients with inferior visual field defects were more impaired compared to those without inferior field defects, who showed no change at all (see Figure 9; T0 is not represented, because of only three patients in group 2).

At T2, the patients with inferior field defects had markedly improved, reaching approximately the values of the patients without inferior defects, but without significance. The effect remained stable at T3. This improvement indicates a positive effect of the EST to QoL.

- **Specific subscales of the GQL-15 questionnaire**

Considering specific subscales of the GQL-15 questionnaire between T1, T2 and T3, group 1 showed a significant improvement (Friedman test, χ^2^(2) = 7.737, *p* = 0.021) in question 8 “avoiding objects on the floor”, but not in the other questions. Post-hoc tests revealed a significant change between T2 and T3 (Wilcoxon signed-rank test, Z = −0.923, *p* = 0.019). After a six-week period without training, the QoL related to avoiding objects was 1.5 (IQR 1–2) at T1 and deteriorated from T2 to T3 from 1.0 (IQR 1–2) to 2.0 (IQR 2–3).

## 4. Discussion

Glaucomatous optic neuropathy affects the retino-geniculo-cortical pathway [33,34,35,36]. This leads to an irreversible and typical loss of visual field, which causes serious impairment in everyday life.

Several recent studies found reduced visual search performance in glaucoma patients [37,38,39,40]. It has been reported that glaucoma patients make more errors in navigating obstacle courses because of the visual field defects [41] and make fewer eye movements towards their visual field defect [42]. In previous studies with patients with hemianopia, it was found that a conscious saccade towards a target in the blind hemifield was characterized by a staircase pattern until the target was detected [11,43], which explains the prolonged reaction times for saccades towards the blind hemifield. An adaptive strategy could develop after a training of this task [43] or after a longer disease duration [11] by applying hypermetric saccades.

In our own previous studies, EST has already proven to be beneficial by compensating for concentric visual field defects in retinitis pigmentosa [6] and in hemianopia after retrochiasmal lesions [5].

The present study focused on potential beneficial effects of EST in advanced glaucoma. We found impaired visual search in patients with scotomas in the 30° visual field, which were binocularly overlapping.

### 4.1. Benefit of Reading Training on Reading Speed (RS)

Reading speed during reading printed texts (RS-print) was reduced at baseline, which was also reported in other studies [8,9,44,45,46]. The RSVP method has been found to be beneficial in our previous studies in patients with central scotoma [13,14], as well as in studies by other authors [12]. After the placebo reading training (PRT), the patients of group 2 showed significant improvement of reading speed on the screen (RS-screen) by 21.3 wpm. Reading speed of printed text (RS-print) improved by 17.12 wpm, which was not statistically significant, but which we consider clinically relevant. Furthermore, the patients of group 2 reached normal RS values at T2, which would be explained by a “consolidation” of the effects of PRT between T1 and T2 and the transfer to everyday life. The high positive correlation between RS-screen and RS-print confirms the positive training effect and shows the transfer of the learned strategy to the real-world reading situation. Therefore, reading training can be recommended for patients with reduced RS.

As expected, reading speed (RS-print) remained unaffected by EST in our study, since reading requires much smaller saccades [19,47] than the explorative saccades that are trained during EST. Group 1 (without reading training) served as a control regarding the reading training, and the fact that RS did not change here indicates the specific effect of PRT.

### 4.2. Benefit of Explorative Saccadic Training (EST) on Reaction Time (RT)

This study has shown a substantial reduction of RT in responses to stimuli on a computer screen, such that patients could find the digits on a computer screen significantly faster after EST.

In addition, during the natural search task (table test), the participants showed significant improvement of RT in the blind and seeing areas, which demonstrated the transfer of the RT improvement from the computer screen to a real-life task.

While there is still no direct evidence supporting an improvement of the visual field as measured by perimetry by any intervention, we hypothesize that the exploratory eye movement training can act as a compensatory method by shifting attention to blind areas and thus training the entire gaze field.

### 4.3. Benefit of Explorative Saccadic Training (EST) on Quality of Life

In our study, we found reduced QoL at baseline using the German version [24] of the GQL-15 questionnaire [20]. The score improved after EST, but did not reach significance. This improvement was observed only in patients with inferior visual field defects and reached approximately the values of the patients without inferior VFD. This shows that patients with inferior defects are much more affected and realize the benefit of the training—even though these differences did not reach significance. This questionnaire includes 15 questions regarding different categories of perception, such as central and near vision, peripheral vision, glare and dark adaptation and outdoor mobility [25,48]. However, EST only trains peripheral vision in the 30° visual field, which is most important for outdoor mobility. Only six questions are related to peripheral vision [20] and only one question of the GQL-15 tests outdoor mobility [20,48]. Therefore, due to the minor focus on the everyday tasks related to peripheral vision, the questionnaire did not sufficiently match the demands of our study, which might explain the lack of significant improvement of the **general** GQL-15 score in our study. However, when planning the study, we decided to use this questionnaire because it had been especially developed for glaucoma patients.

Furthermore, a longer or more continuous training period than the six weeks in our study may increase the training effect. This is supported by the finding that at level 3 of EST, there is still significant decrease of reaction time for all patients (see Figure 3 and Table 2), which indicates a potential for further improvement.

The GQL-15 questionnaire focuses only on the physical effects of glaucoma [25] and does not test the subjective well-being of patients, such as anxiety and depression [49]. For determination of patients’ psychological status, specific questionnaires can be used, as we did in our previous studies with patients with age-related macular degeneration in order to assess the effect of visual rehabilitation [13,32]. However, during the study design phase, we decided to favour the GQL-15 because it had been specially developed for patients with glaucoma [50] and because of its better compatibility with the daily routine in an ophthalmologists’ office.

A possible indirect indication of an eventual improvement of QoL could be the compliance (acceptance rate) of the training program: Every patient at the end of the study was asked to decide if he or she would continue to train, and no recommendation was given. It turns out that half of the patients (12 of 24) decided to continue using the program after 3 months, which could indicate a beneficial effect of EST in some patients.

### 4.4. Strengths and Limitations

We acknowledge that our study had several limitations. Binocular perimetry for verification of the estimated binocular overlapping absolute scotoma was not available. A larger sample size might have allowed analysis of subgroups and would have provided more statistical power. In addition, a later follow-up might have captured more of the impact of EST on quality of life. Future studies should examine these questions in a larger cohort.

The strengths of the study are its randomized placebo-controlled and prospective character as well as the strict inclusion and exclusion criteria. To the best of our knowledge, this is the first compensatory saccade training study to investigate visual search performance and reading training in patients with glaucoma. The focus on the effects of EST on two abilities that are decisive for everyday life, namely exploration and reading, is a further strength. The measurement and comparison of RT and reading speed both on a computer screen and in a real-life situation show the transfer to daily routine. The fact that the training programs can be used at home makes them particularly practice-orientated.

### 4.5. Hypotheses

Regarding our hypotheses, we can state the following:

- Hypotheses 1–3 were confirmed (EST improved RT on a screen and in a natural search task, PRT did not influence RT in search tasks and was therefore appropriate as placebo control group);
- Hypothesis 4 has to be rejected: PRT **did** improve RS.

## 5. Conclusions and Outcome

The outcome of this randomized controlled study showed the following:

1. Glaucoma patients with binocular overlapping visual field defects improved their reaction time during exploration, significantly in their blind area;
2. Reading training improved their reading performance;
3. These compensatory strategies were transferred to everyday life.

## Figures and Tables

**Table 1:** Clinical data at baseline. Group 1 had one type of training; group 2 had two types. BCVA (best corrected visual acuity), IOP (intraocular pressure), MD (mean defect).

	Group 1	Group 2
number of patients	14 (5 women)	11 (3 women)
age (years)	75 (62.7–80.7)	72 (66–80)
open angle glaucoma	10	9
pseudoexfoliative glaucoma	2	1
normal tension glaucoma	2	1
topical antiglaucoma medication (classes)	3 (2–3)	3 (2–3)
glaucoma surgery	6	6
cataract surgery	7	3
BCVA best eye	0.8 (0.63–1.0)	1.0 (0.8–1.0)
IOP (mmHg)	11 (10–13)	13 (10.3–15)
perimetric MD (dB)	15.2 (11.9–19.3)	14.1 (10.4–15.7)

**Table 2:** Reaction time (RT) during explorative saccadic training (EST)—level 1, level 2, level 3 at start and end—group 1, 2, all patients (median and interquartile range (IQR).

Group	Level 1		Level 2		Level 3	
EST	Start	End	Start	End	Start	End
**1**	3.14 s (2.50–12.62)	2.54 s (1.97–5.50)	2.60 s (1.96–4.85)	2.32 s (1.79–4.10)	3.10 s (2.11–4.91)	2.85 s (2.02–4.03)
	Z = −3.180, *p* = 0.001		Z = −3.107, *p* = 0.002		Z = −1.789, *p* = 0.074	
**2**	2.79 s (2.01–6.71)	2.32 s (1.80–4.47)	2.35 s (1.78–4.22)	2.18 s (1.72–3.54)	2.51 s (1.92–4.89)	2.46 s (1.95–3.31)
	Z = −2.134, *p* = 0.033		Z = −2.667, *p* = 0.008		Z = −1.245, *p* = 0.213	
All patients	2.86 s (2.36–8.58)	2.52 s (1.90–4.46)	2.47 s (1.90–4.05)	2.32 s (1.79–3.57)	2.53 s (2.07–4.49)	2.48 s (2.04–3.65)
	Z = −3.771, *p* < 0.001		Z = −4.103, *p* < 0.001		Z = −2.274, *p* = 0.023	
group comparison between 1 & 2	U = 53 *p* = 0.303	U = 65 *p* = 0.733	U = 68 *p* = 0.647	U = 76 *p* = 0.979	U = 68 *p* = 0.647	U = 74 *p* = 0.893

Comparison between groups 1 and 2 at start and end of all three levels. Z = value of the Wilcoxon signed-rank test; *p* = probability value. U = Mann–Whitney U Test statistic.

**Table 6:** Reading speed (RS-print) in group 1 and 2 at T0, T1 and T2 (for details see text).

RS-Print (Wpm)	T0	T1	T2
**group 1**	-	**145.40 wpm** (122.76−174.80)	**148.11 wpm** (129.98−163.30)
Wilcoxon signed-rank test	-	Z = 50.0, *p* = 0.753	
**group 2**	**144.56 wpm** (76.35−170.29)	**161.68 wpm** (78.95−182.29)	**162.70 wpm** (93.84−179.59)
Friedman test	χ^2^(2) = 2.600, *p* = 0.273		

## Data Availability

Due to the sensitive data and small number of patients, the raw data are only available after a separate query.
